# Supplementary material for: Evaluating structure selection in the hydrothermal growth of FeS2 pyrite and marcasite
Source: Nat Commun. 2016 Dec 14;7:13799. doi: 10.1038/ncomms13799 (PMC5171653; doi:10.1038/ncomms13799)
Supplement: Supplementary Information — Supplementary figures, supplementary tables and supplementary references. [file ncomms13799-s1.pdf]

SUPPLEMENTARY FIGURES

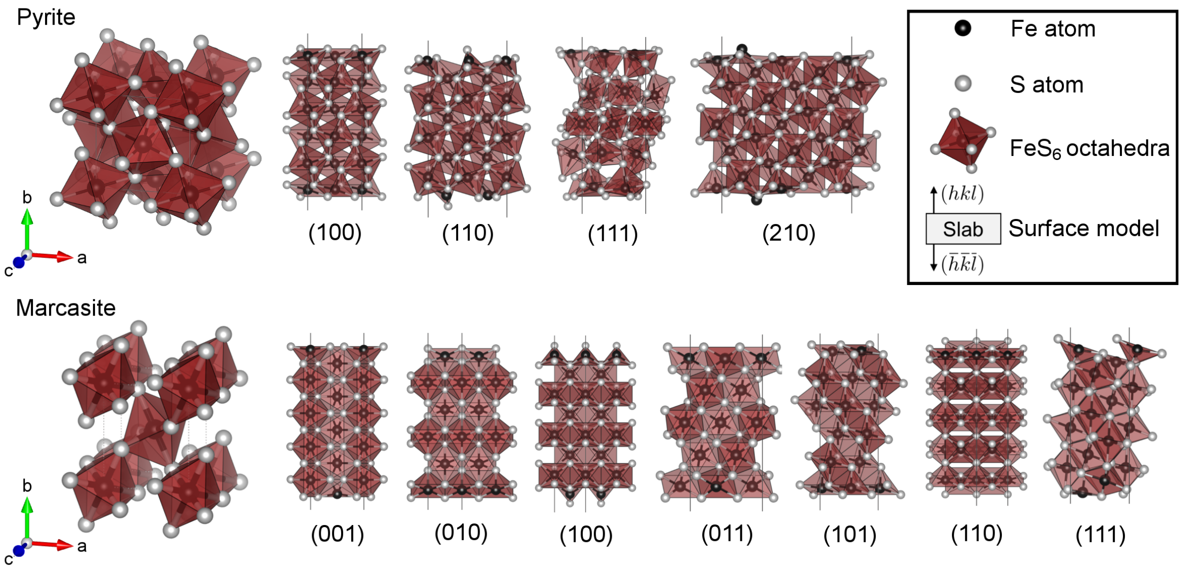

Supplementary Figure 1: Structure of FeS_2 bulk and surface slab models. The structures of FeS_2 pyrite (top) and marcasite (bottom) bulk, as well as the relaxed surface slab models of the significant crystallographic facets of both phases. The surface structures represent 2D periodic slabs of a thickness large enough such that the few middle layers of the slab are energetically "bulk-like", while the top and bottom layers capture the structure and energetics of the (hkl) and $(\bar{h}\bar{k}\bar{l})$ facets respectively.

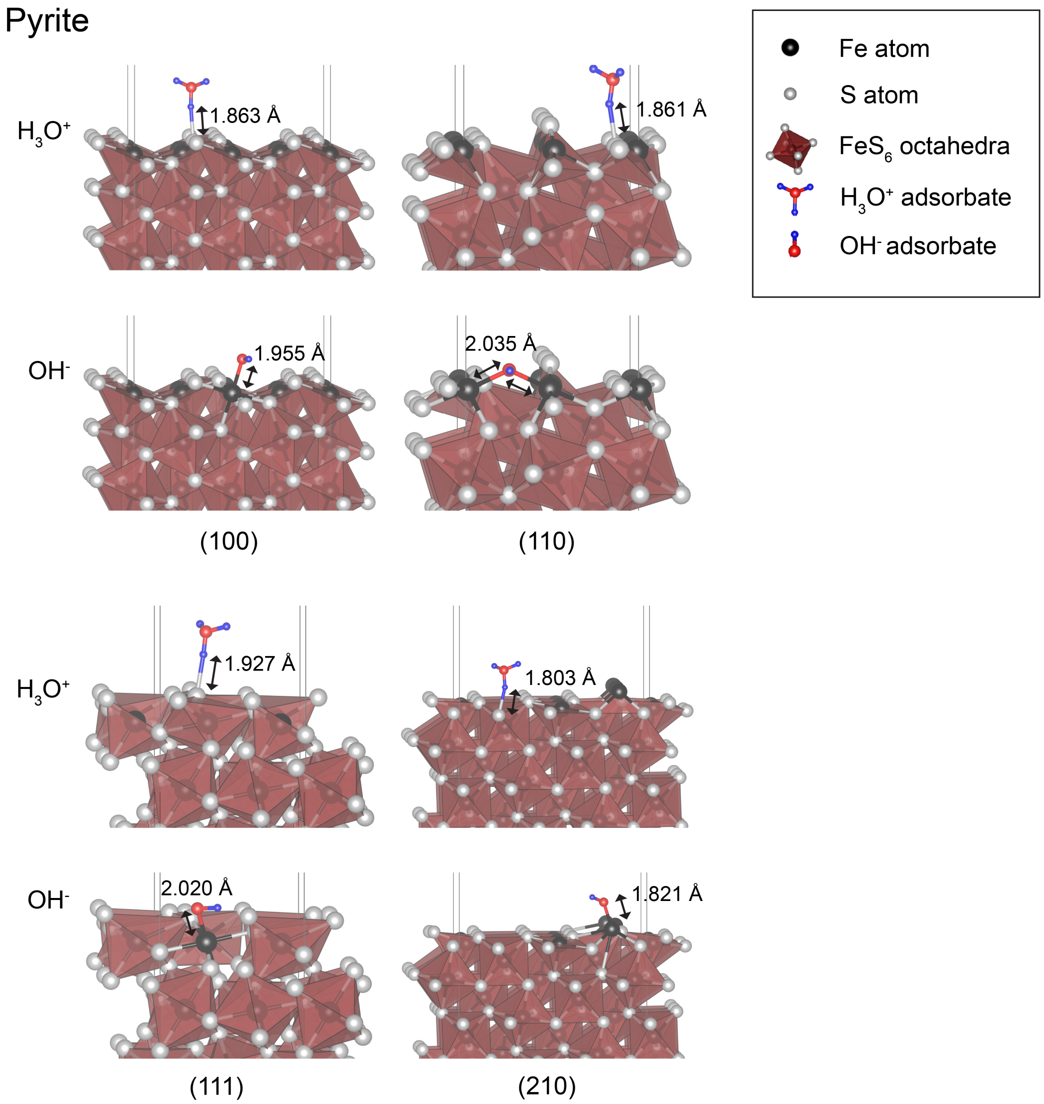

Supplementary Figure 2: Relaxed dilute adsorption geometries on pyrite. The relaxed geometries of H_3O^+ and OH^- adsorbed onto the major facets of FeS_2 pyrite. In all cases, the charge of the adsorbate is compensated by a countercharge in the vacuum, and replicated on the opposite side of the slab (not shown) to ensure that the calculation cell has no net dipole, following the calculation methodology described in the main text.

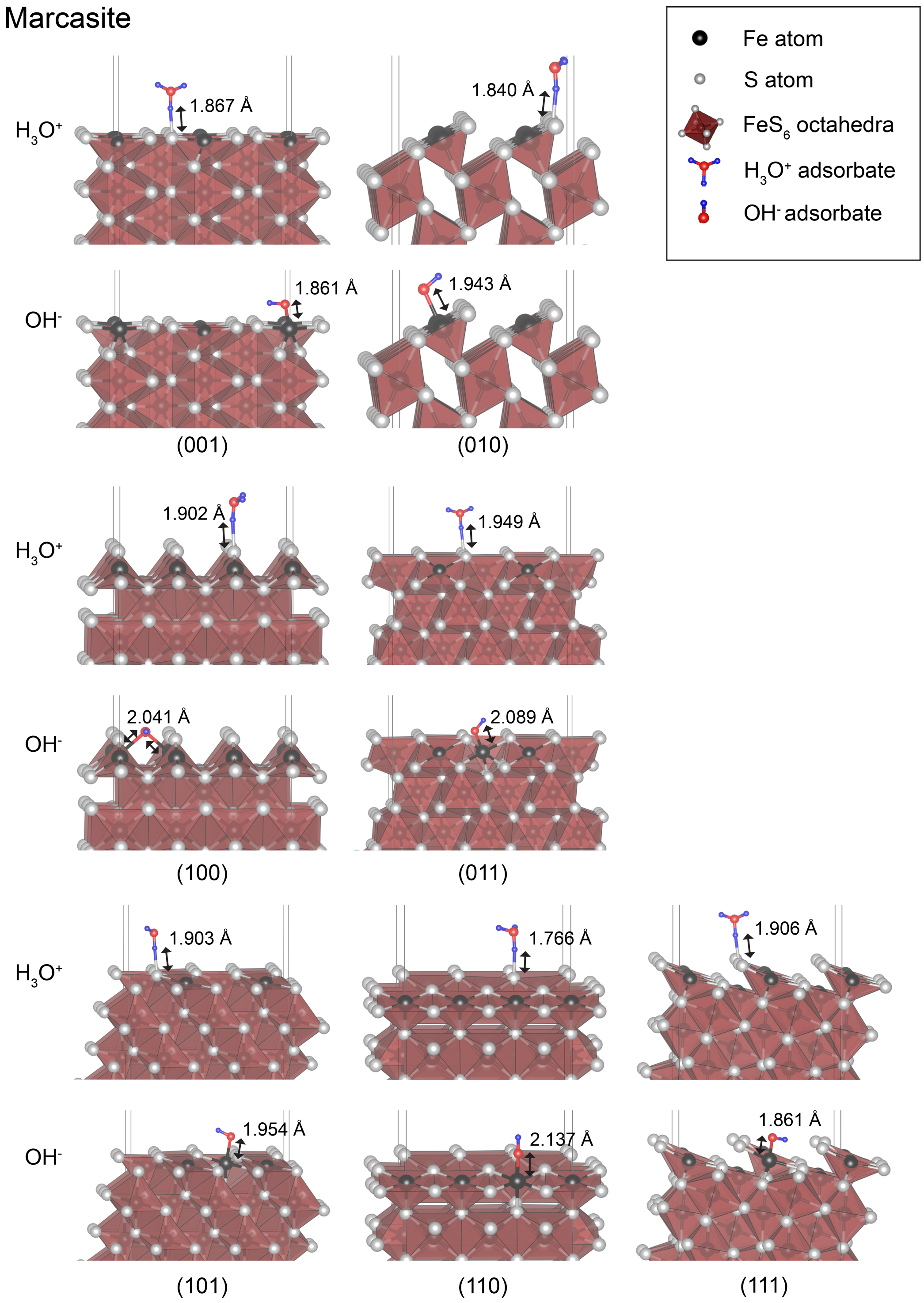

Supplementary Figure 3: Relaxed dilute adsorption geometries on marcasite. The relaxed geometries of H_3O^+ and OH^- adsorbed onto FeS_2 marcasite. In all cases, the charge of the adsorbate is compensated by a countercharge in the vacuum, and replicated on the opposite side of the slab (not shown) to ensure that the calculation cell has no net dipole, following the calculation methodology described in the main text.

SUPPLEMENTARY TABLES

Supplementary Table I: Bulk thermodynamics of pyrite and marcasite.
Calculated and experimental bulk parameters for pyrite and marcasite phases of FeS_2, as well as the most important crystallographic facets of both phases. References: [1–7]

**Phase**	**Lattice (Å)** (a, b, c)	$\mathbf{H_f}$ (meV/f.u.)	**Facets**
Pyrite (Pa3)	Calc: 5.329, 5.329, 5.329<br>Exp: 5.416, 5.416, 5.416	Calc: 0<br>Exp: 0	(100), (110), (111), (210)
Marcasite (Pnnm)	Calc: 4.371, 5.339, 3.347<br>Exp: 4.433, 5.426, 3.389	Calc: 8.3<br>Exp: 43 ± 2	(100), (010), (001), (110)<br>(101), (011), (111)

SUPPLEMENTARY NOTES

Supplementary Note 1: Derivation of the reference entropy of ions in solution

First, consider H_3O^+ in a solution at a set pH. By the definition of pH, the chemical potential of H_3O^+ can be written as

$$\mu_{H_3O^+} = \mu^0_{H_3O^+} + k_B T \log a_{H_3O^+} = \mu^0_{H_3O^+} - 2.3 k_B T \text{ pH}$$

where the reference state chemical potential $\mu^0_{H_3O^+}$ corresponds to a 1M solution of H_3O^+ at standard state conditions (temperature T_0). By the ideal solution model, this reference chemical potential can be written as

$$\mu^0_{H_3O^+} = h_{H_3O^+} + k_B T_0 \ln \frac{1}{M_w} = h_{H_3O^+} - k_B T_0 \ln M_w$$

Thus, the chemical potential of H_3O^+ at any pH is given by

$$\mu_{H_3O^+} = h_{H_3O^+} - k_B T_0 \ln M_w - 2.3 k_B T \text{ pH}$$

giving an entropy of

$$s_{H_3O^+} = -\frac{\mu_{H_3O^+} - h_{H_3O^+}}{T} = k_B \frac{T_0}{T} \ln M_w + 2.3 k_B \text{ pH}$$

The chemical potential of OH^- is set by the equilibrium of H_3O^+, OH^- and H_2O:

$$\mu_{OH-} = 2\mu_{H_2O} - \mu_{H_3O^+} = 2h_{H_2O} - h_{H_3O^+} + k_B T_0 \ln M_w + 2.3 k_B T \text{ pH}$$

Once again, we can solve for the entropy of OH^- to yield:

$$s_{OH^-} = -\frac{\mu_{OH^-} - h_{OH^-}}{T} = \frac{h_{OH^-} + h_{H_3O^+} - 2h_{H_2O}}{T} - k_B \frac{T_0}{T} \ln M_w - 2.3 k_B \text{ pH} =$$

$$= \frac{\Delta h^0_w}{T} - k_B \frac{T_0}{T} \ln M_w - 2.3 k_B \text{ pH}$$

Supplementary Note 2: Example calculation of total surface energy, accounting for adsorption

To illustrate the interplay between the terms introduced in the thermodynamic model and their relative importance, we derive the adsorbed surface energies for two example surfaces at pH 5.5.

In pyrite, under these conditions, we find that the dominant surface is (210), adsorbed with OH^- ions. The energy of the clean (210) surface is $E_{slab}^{vac}/2A = 1.82$ J m^{-2}, but this energy is significantly reduced by hydration and adsorption. The number and geometry of OH^- adsorption sites can be estimated from the "missing" S atoms which would have coordinated the Fe on the surface. These sites occur at a density of $N_{slab}^{sites} = 0.0787$ Å^{-2}. For each of these sites, the energy of mean–field H_2O adsorption is calculated to be ΔE_{slab}^{solv} = -0.596 eV per site. The energy of the competing OH^- adsorption (at pH 5.5) is calculated to be $\Delta E_{OH^-}^{ads,\infty}$ = -0.813 eV per OH^-, where $\Delta E_{OH^-}^{ads,\infty}$ accounts for adsorbate–solid interactions referenced to the chemical potential of the OH^- ion in solution at pH = 5.5, but not adsorbate–adsorbate interactions or configurational entropy. To find the total energy of the adsorbed surface, we minimize the total energy of the interface with respect to OH^- coverage, combining the properly referenced adsorbate–solid interactions in the form of $\Delta E_{OH^-}^{ads,\infty} - \Delta E_{slab}^{solv}$ = -0.217 eV, the adsorbate–adsorbate interactions in the form of the Debye–Huckel term V^{el}, and the configurational entropy. We find that the optimum occurs at 9% coverage, where the Debye-Huckel repulsion contributes 0.140 eV per OH^-, and the entropy contributes -0.138 eV per OH^- at the preset 473K temperature. Put together, the adsorption lowers the surface energy by 0.024 J m^{-2} with respect to the pure hydrated surface (1.069 J m^{-2}), giving a final surface energy of 1.045 J m^{-2}.

In marcasite, one important OH^-–stabilized facet is the (110) surface. The vacuum energy of this surface is 1.66 J m^{-2}, brought down to 1.19 J m^{-2} by hydration. Following the same broken–bond–counting metric as before, we estimate the density of OH^- adsorption sites to be 0.086 Å^{-2}, with $\Delta E_{OH^-}^{ads,\infty} - \Delta E_{slab}^{solv}$ = -0.770 eV per OH^-. We find that the optimal coverage is 16%, with the Debye-Huckel repulsion term contributing 0.333 eV per OH^- and configurational entropy contributing -0.111 eV per OH^-. Thus, OH^- adsorption lowers the surface energy by 0.124 J m^{-2}, yielding a final surface energy of 1.061 J m^{-2}.

SUPPLEMENTARY REFERENCES

[1] Gronvold, F. & Westrum Jr., E. F. Heat capacities of iron disulfides: thermodynamics of marcasite from 5 to 700 K, pyrite from 300 to 780 K, and the transformation of marcasite to pyrite. *The Journal of Chemical Thermodynamics*, **8**, 1039 – 1048 (1976).

[2] Lowson, R. T. Aqueous oxidation of pyrite by molecular oxygen. *Chemical Reviews*, **82**, 461–497 (1982).

[3] Murowchick, J. B. & Barnes, H. Marcasite precipitation from hydrothermal solutions. *Geochimica et Cosmochimica Acta*, **50**, 2615–2629 (1986).

[4] Schmokel, M. S. *et al.* Atomic properties and chemical bonding in the pyrite and marcasite polymorphs of FeS_2: a combined experimental and theoretical electron density study. *Chem. Sci.*, **5**, 1408–1421 (2014).

[5] Hung, A., Muscat, J., Yarovsky, I. & Russo, S. P. Density-functional theory studies of pyrite FeS_2 (100) and (110) surfaces. *Surface Science*, **513**, 511 – 524 (2002).

[6] Hung, A., Muscat, J., Yarovsky, I. & Russo, S. P. Density-functional theory studies of pyrite FeS_2 (111) and (210) surfaces. *Surface Science*, **520**, 111 – 119 (2002).

[7] Richards, R. P., Clopton, E. L. & Jaszczak, J. A. Pyrite and marcasite intergrowths from northern illinois. *Mineralogical Record*, **26**, 129 (1995).
